# Conscious prone positioning in nonintubated COVID-19 patients with acute respiratory distress syndrome: systematic review and meta-analysis

**DOI:** 10.62675/2965-2774.20240176-en

**Published:** 2024-03-25

**Authors:** Gustavo Adolfo Vásquez-Tirado, Edinson Dante Meregildo-Rodríguez, Martha Genara Asmat-Rubio, María José Salazar-Castillo, Claudia Vanessa Quispe-Castañeda, María del Carmen Cuadra-Campos

**Affiliations:** 1 Universidad Privada Antenor Orrego Escuela de Medicina Trujillo Peru Escuela de Medicina, Universidad Privada Antenor Orrego - Trujillo, Peru.; 2 Universidad César Vallejo Escuela de Medicina Trujillo Peru Escuela de Medicina, Universidad César Vallejo - Trujillo, Peru.; 3 Universidad Privada Antenor Orrego Escuela de Posgrado Trujillo Peru Escuela de Posgrado, Universidad Privada Antenor Orrego - Trujillo, Peru.

**Keywords:** COVID-19, Coronavirus infections, Prone position, Patient positioning, Hospital mortality, Mortality

## Abstract

**Objective:**

To systematically review the effect of the prone position on endotracheal intubation and mortality in nonintubated COVID-19 patients with acute respiratory distress syndrome.

**Methods:**

We registered the protocol (CRD42021286711) and searched for four databases and gray literature from inception to December 31, 2022. We included observational studies and clinical trials. There was no limit by date or the language of publication. We excluded case reports, case series, studies not available in full text, and those studies that included children < 18-years-old.

**Results:**

We included ten observational studies, eight clinical trials, 3,969 patients, 1,120 endotracheal intubation events, and 843 deaths. All of the studies had a low risk of bias (Newcastle-Ottawa Scale and Risk of Bias 2 tools). We found that the conscious prone position decreased the odds of endotracheal intubation by 44% (OR 0.56; 95%CI 0.40 - 0.78) and mortality by 43% (OR 0.57; 95%CI 0.39 - 0.84) in nonintubated COVID-19 patients with acute respiratory distress syndrome. This protective effect on endotracheal intubation and mortality was more robust in those who spent > 8 hours/day in the conscious prone position (OR 0.43; 95%CI 0.26 - 0.72 and OR 0.38; 95%CI 0.24 - 0.60, respectively). The certainty of the evidence according to the GRADE criteria was moderate.

**Conclusion:**

The conscious prone position decreased the odds of endotracheal intubation and mortality, especially when patients spent over 8 hours/day in the conscious prone position and treatment in the intensive care unit. However, our results should be cautiously interpreted due to limitations in evaluating randomized clinical trials, nonrandomized clinical trials and observational studies. However, despite systematic reviews with meta-analyses of randomized clinical trials, we must keep in mind that these studies remain heterogeneous from a clinical and methodological point of view.

## INTRODUCTION

Coronavirus disease 2019 (COVID-19) was first detected in Wuhan (China), after which it spread throughout the world. Of all infected patients with severe acute respiratory syndrome coronavirus 2 (SARS-CoV-2), approximately 20% have mild disease that may or may not complicate hypoxemia, thus requiring hospitalization.^(1,2)^ The most severe manifestation of COVID-19 is acute respiratory failure (ARF) due to acute respiratory distress syndrome (ARDS).^(3)^ Approximately five percent of COVID-19 patients require admission to the intensive care unit (ICU) and invasive mechanical ventilation (IMV) due to ARF and ARDS.^(4-7)^

The prone ventilation strategy with IMV has been practiced since 1970 in patients with atelectasis and decreased lung compliance. This technique allows for the recruitment of the pulmonary posterobasal zones, thus improving pulmonary oxygenation^(2,8,9)^ and the ventilation/perfusion ratio of these lung segments.^(4,6,10-12)^ The prone position is one of the few strategies shown to decrease mortality in ARDS. However, the time in the prone position and the cycle duration need to be individualized for each patient, and their specific parameters are not precise.

The experience gained from the prone position strategy in mechanically ventilated patients with ARDS without COVID-19 has been extrapolated to awake patients with COVID-19, which has shown promising results.^(4,13)^ Additionally, during the COVID-19 pandemic, it was used as a ventilatory strategy in awake, nonintubated COVID-19 patients. It was also administered to intubated patients with other non-COVID-19 respiratory diseases. According to current evidence, it seems clear that the prone position improves gasometric oxygenation.^(14)^ However, whether this strategy decreases the risk of endotracheal intubation (ETI) or mortality^(15)^ in conscious prone position (CPP), nonintubated COVID-19 patients is unclear.

To date, there is a body of evidence regarding the relationship between CPP and its impact on the risk of ETI and mortality, as explored in randomized clinical trials (RCTs) and systematic reviews with meta-analyses (SR-Ms).^(16-19)^ Current evidence suggests no significant reduction in mortality associated with CPP therapy. It is important to note that the SR-Ms of the RCTs in question primarily consider statistical heterogeneity, thus overlooking potential methodological and clinical variations that may contribute to this observed outcome.

Concerning mortality, the exploration of other subgroups, such as extended prone positioning duration and the care setting (e.g., within or outside the ICU), could play a pivotal role in elucidating this association.^(18)^

## METHODS

### Information sources and search strategy

We previously registered the protocol in PROSPERO (CRD42021286711). In addition, we performed an exhaustive search of primary articles (cross-sectional, cases and controls, cohorts and randomized clinical trials) in four databases (PubMed, Embase, Scopus, and Web of Science) and gray literature (Mednar and WorldWideScience) from inception up to December 31, 2022. We followed a PECO/PICO strategy (population: "nonintubated COVID-19 patients with acute respiratory failure", intervention: "conscious prone positioning", control: "standard treatment", outcome: "endotracheal intubation" OR "mortality"). By using Boolean connectors, we combined free and controlled vocabulary (MESH and Emtree headings) terms (Table 1S - Supplementary material). During the performance of the present study, no amendments to the protocol were made.

### Study selection

**Figure 1:**
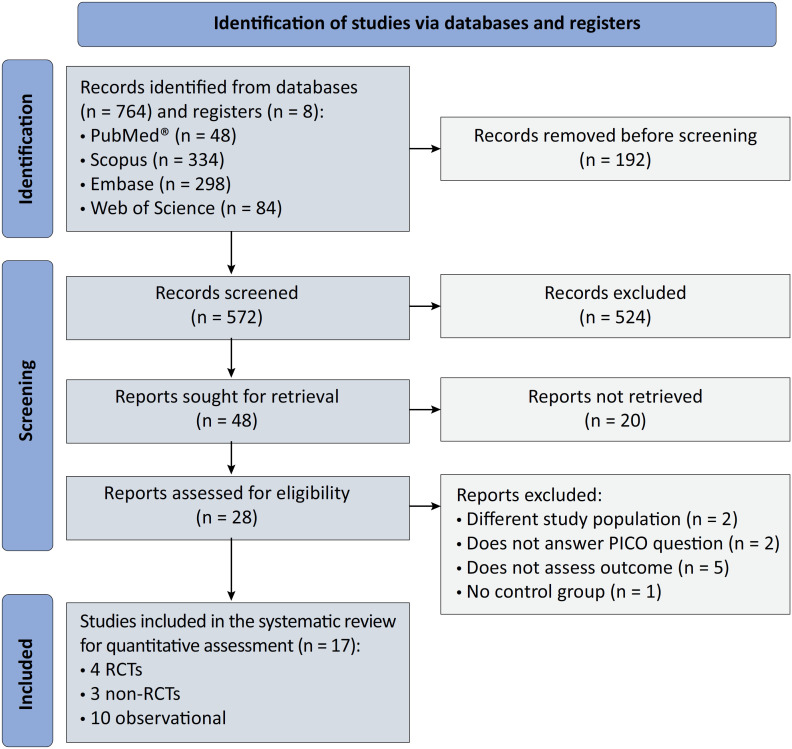
PRISMA 2020 flow diagram of the selection process of the primary included studies.

We collected documents in full text and abstracts. There was no limit by date or the language of publication. We excluded case reports, case series, studies not available in full text, and those that included children < 18-years-old. Three independent blinded researchers assessed the papers. Discrepancies were resolved by consensus or as a last resort by a fourth reviewer who acted as a referee. Figure 1 shows the selection process.

RCT - randomized controlled trial.

### Data extraction and risk of bias assessment

We analyzed the articles according to our PECO/PICO strategy and the inclusion and exclusion criteria. Using a spreadsheet, we extracted and registered data concerning authors, year of publication, country of publication, type of study, and the number of patients, controls, and events (ETI and mortality).

### Data synthesis and analysis

We pooled odds ratios (ORs) by using the Mantel-Haenszel method in the meta-analysis. We examined two primary outcomes: the odds of orotracheal intubation and mortality. In addition, due to the fact that heterogeneity was substantial (I2 > 40%), we performed random effects models and subgroup analysis according to the patient's daily time > 8 hours/day or less, based on the average daily total time of CPP for most patients reported in the studies and the patients’ care locations (ICU or no ICU).^(18)^

We assessed the study quality by using the Cochrane Risk of Bias 2 tool 2 (RoB 2) for RCTs,^(20)^ Risk of Bias in Non-randomised Studies of Interventions (ROBINS-I) for non-RCTs and Newcastle-Ottawa Scale (NOS) for observational studies,^(21)^ as well as the risk of publication bias by using funnel plots.

Two independent reviewers examined the certainty of the evidence of the study outcomes for each study outcome based on the Grading of Recommendations Assessment, Development and Evaluation (GRADE) criteria. Any discrepancy between the review authors was resolved by discussion with the leading researcher.

## RESULTS

### Search results and study characteristics

We identified 764 records in the primary systematic search and 8 records in the secondary examination. After eliminating duplicates, 572 records remained for review in the title and abstract. Subsequently, we excluded 544 records. Finally, we assessed 28 records in full text in the qualitative synthesis (Table 1).^(1,19-37)^ Afterwards, 17 articles remained for the meta-analysis, of which 10 were observational studies, 4 were RCTs and 3 were non-RCTs (Figure 1).

**Table 1:** General characteristics of the included studies

Author	Country/Study	Participants	Outcomes
Coppo et al.^(6)^	Italy UC, PCS	n = 46 patients. Both sexes	ETI in 18 patients, five deaths unrelated to the procedure
Rosén et al.^(10)^	Sweden MC, RCT	n = 75 were randomized, 39 in the SC Group and 36 in the CPP Group. Both sexes	13 patients needed ETI in the SC Group *versus* 12 patients in the CPP Group (HR 1.01; 95%CI 0.46 - 2.21)
Tonelli et al.^(13)^	Italy MC, RCS	n = 114 patients, 76 in the SC and 38 in the CPP Group. Both sexes	22 patients died (17 in SC Group and 5 in CPP Group); 37 patients needed ETI (7 in CPP Group and 30 in SC Group)
Sryma et al.^(14)^	India UC, non-RCT	n = 45 subjects (30 cases and 15 controls). Both sexes.	The need for IMV was higher in the Control Group (33.3%) *versus* Prone Group (6.7%).
Ferrando et al.^(33)^	Spain and Andorra MC, PCS	n = 199 patients on HFNC therapy. Both sexes	82 patients needed ETI (HFNC Group 60, HFNC + CPP Group 22); 17 died in the HFNC Group, and eight in the HFNC + CPP Group
Dubosh et al.^(34)^	USA UC, PCS	n = 22 patients. Both sexes.	7 patients needed ETI (5 patients in the first 48 hours, 2 after the next 48 hours), 2 patients died
Kaur et al.^(35)^	USA UC, RCT	n = 125 patients. Of them, 92 received early CPP, and 33 received late CPP. Both sexes	The early CPP Group had lower mortality than the late CPP Group (26% *versus*. 45%, p = 0.039), without a difference in ETI rate. However, advanced age, ETI, longer time to initiate CPP and hydrocortisone use were associated with increased mortality
Ehrmann et al.^(36)^	Europe, North America and South America MC, RCT	n = 1,126 patients, CPP 567, SC 559. Included in the ITT analysis 1,121 patients. Both sexes	Treatment failure in 40% of patients in the CPP Group and 46% of patients in the SC Group (RR 0.86; 95%CI 0.75 - 0.98). HR for ETI 0.75 (95%CI 0.62 - 0.91), HR for mortality 0.87 (95%CI 0.68 - 1.11) with CPP compared with SC at 28 days of enrollment
Perez-Nieto et al.^(37)^	México, and Ecuador. MC, RCS	n= 827 nonintubated patients in the CPP (n = 505) and SC (n = 322) Groups. Both sexes	Fewer patients in the CPP Group needed ETI (23.6% *versus*. 40.4%) or died (19.8% *versus*. 37.3%)
Dueñas-Castell et al.^(38)^	Colombia UC, RCS	n = 212 patients with SC and CPP. Both sexes.	Overall mortality 34% (73/212)
Vianello et al.^(39)^	Italy UC, PCS	n = 93 patients were included in the study. Both sexes	CPP was feasible and safe in 50 patients. Sixteen patients received ETI, and 27 escalated respiratory support. The mortality rate was 9/93. In 41/50 of subjects who passed the trial and underwent CPP, there was clinical benefit and survival without escalation of therapy
Altinay et al.^(40)^	Turkey UC, RCS	n = 72. CPP Group (n = 49), analyzed (n = 25). SC Group (n = 23), analyzed (n = 23). Both sexes	CPP Group: 9 patients died, and eight needed ETI (p = 0.020). SC Group: 16 patients died, and 19 needed ETI (p = 0.001)
Jayakumar et al.^(41)^	India MC, RCT	n= 60 patients. CPP 30, SC: 30. Both sexes	CPP Group: 3 patients died, and four needed ETI. SC Group: 2 patients died, and 4 needed ETI
Solverson et al.^(42)^	Canada MC, RCS	n = 17 patients. Both sexes. ICU 12, hospital ward 5	7 patients needed ETI and IMV; 2 patients died in the ICU after a course of IMV
Gad et al.^(43)^	Egypt MC, RCS	n = 30 patients; CPP 15; NIV 15	6 patients needed IMV (3 in the CPP Group and 3 in the NIV Group); these same six patients died
Burton-Papp et al.^(44)^	USA UC, RCS	n = 81 patients. A total of 20 patients received CPP in conjunction with NIV	7 patients were intubated; no patient died
Tatlow et al.^(45)^	Australia UC, RCS	n = 13 patients. Both sexes	7 patients died
Bahloul et al.^(46)^	Túnez UC, PCS	n = 21 patients. Both sexes. CPP Group 21, SC Group 17	7 patients died in the CPP Group and 5 in the SC Group; 9 needed IMV. CPP was not associated with a reduction in mortality or IMV rate (p > 0.05 for both)
Pierucci et al.^(47)^	Italy UC, PCS	n = 32 patients, CPP 16, NIV 16. Both sexes	5 patients were intubated (3 in the SC Group and 2 in the CPP), and 3 in the SC Group died (the same ones needed ETI)
Winearls et al.^(48)^	UK UC, RCS	n = 24 patients with CPAP. Both sexes	1 patient had invasive ventilation and 4 patients died
Musso et al.^(49)^	Italy UC, Non-RCT	n = 243 patients; CPP Group 81, SC Group 162. Both sexes	69 patients died, 59 in the SC Group and 10 in the CPP Group; 52 patients needed ETI, 44 in the control Group, and 8 in the CPP Group
Aisa et al.^(50)^	Ireland UC, PCS	n = 50 patients	7 patients were intubated (14%). CPP was feasible in 41 (82%) patients, and 38 (76%) patients reported good tolerance
Althunayyan et al.^(51)^	Saudi Arabia UC, PCS	n = 49 patients. Both sexes	6 patients were intubated (12.2%) and 7 patients died (14.3%)
Qian et al.^(52)^	USA MC, Non-RCT	n = 501 patients assigned 1:1 to either CPP Group or SC Group. Both sexes	CPP Group: 31 patients were intubated and 56 patients died. SC Group: 30 were intubated, 47 died.
Fralick et al.^(53)^	Canada, USA MC, RCT	n = 248 patients; CPP Group 126, SC Group 122. Both sexes	2 patients died, one from CPP and one from the SC Group
Barker et al.^(54)^	UK UC, RCCS	n = 20 patients; CPP Group 10 patients, SC Group 10 patients. Both sexes	6 (60%) patients needed IMV in the CPP Group and 5 (50%) in the SC Group; 1 (10%) died in the CPP Group and 4 (40%) in the SC Group.
Esperatti et al.^(55)^	Argentina MC, PCS	n = 335 patients; CPP Group 187, SC Group 148. Both sexes	44 patients in the CPP Group (23%) and 79 (53%) in the SC Group were intubated; 2 patients died, one from each group.
Kumar et al.^(56)^	India UC, PCS	n = 67 patients	NR

UC - unicenter, PCS - prospective cohort study; ETI - endotracheal intubation; MC - multicenter; RCT - randomized controlled trial; SC - standard care; CPP - conscious prone position; HR - hazard ratio; 95%CI - 95% confidence interval; RCS - retrospective cohort study; IMV - invasive mechanical ventilation; HFNC - high-flow nasal cannula; ITT – intent-to-treat; ICU - intensive care unit; NIV - noninvasive mechanical ventilation; RCCS - retrospective case-control study; NR - not reported.

This study collected 3,969 participants, 1,120 ETI events, and 843 deaths. Studies were developed worldwide during the COVID-19 pandemic (Table 1) until December 31, 2022.

### Risk of bias in studies

**Figure 2:**
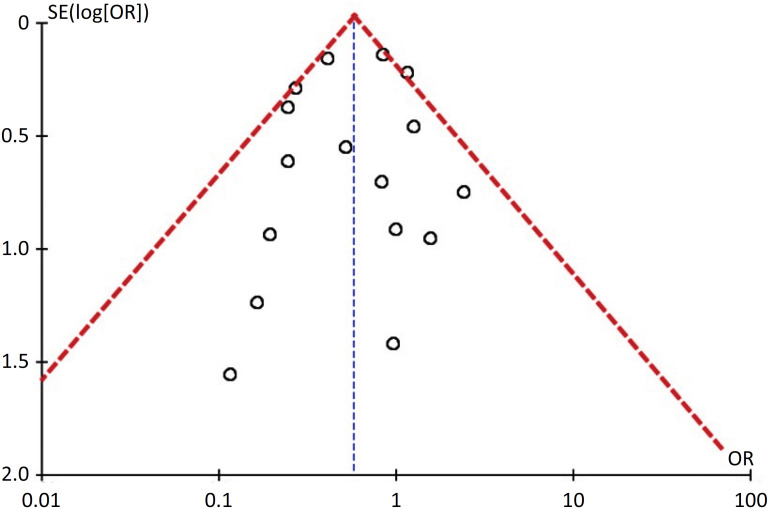
Funnel plot of the effect of continuous prone positioning on the odds of mortality.

All of the included studies had a low risk of bias (Table 2); for this reason, 3 non-RCTs were considered RCT analyses. The funnel plot does not suggest a risk of publication bias among the included studies (Figure 2).

**Table 2:** Bias assessment of the included primary studies

Study	Type study	Site of attendance	Tool	Conclusion
Rosén et al.^(10)^	RCT	ICU and medical ward	ROB 2	Low risk
Tonelli et al.^(13)^	Retrospective cohort study	ICU	NOS	Low risk
Sryma et al.^(14)^	Non-RCT	No data	ROBINS-I	Low risk
Ferrando et al.^(33)^	Prospective cohort study	ICU	NOS	Low risk
Ehrmann et al.^(36)^	RCT	ICU and medical ward	ROB 2	Low risk
Perez-Nieto et al.^(37)^	Retrospective cohort study	ICU and medical ward	NOS	Low risk
Altinay et al.^(40)^	Retrospective cohort study	ICU	NOS	Low risk
Jayakumar et al.^(41)^	RCT	ICU	ROB 2	Low risk
Gad et al.^(43)^	Retrospective cohort study	ICU	NOS	Low risk
Burton-Papp et al.^(44)^	Retrospective cohort study	ICU	NOS	Low risk
Bahloul et al.^(46)^	Prospective cohort study	ICU	NOS	Low risk
Pierucci et al.^(47)^	Prospective cohort study	Medical ward	NOS	Low risk
Musso et al.^(49)^	Non-RCT	ICU	ROBINS-I	Low risk
Qian et al.^(52)^	Non-RCT	ICU and medical ward	ROBINS-I	Low risk
Fralick et al.^(53)^	RCT/MW	Medical ward	ROB 2	Low risk
Barker et al.^(54)^	Retrospective case control	ICU	NOS	Low risk
Esperatti et al.^(55)^	Prospective cohort study	ICU	NOS	Low risk

RCT - randomized controlled trial; ICU - intensive care unit; ROB 2 - Risk of Bias 2; NOS - Newcastle-Ottawa Scale; ROBINS-I - Risk of Bias in Non-randomised Studies of Interventions; MW - medical ward.

### Risk of endotracheal intubation

**Figure 3:**
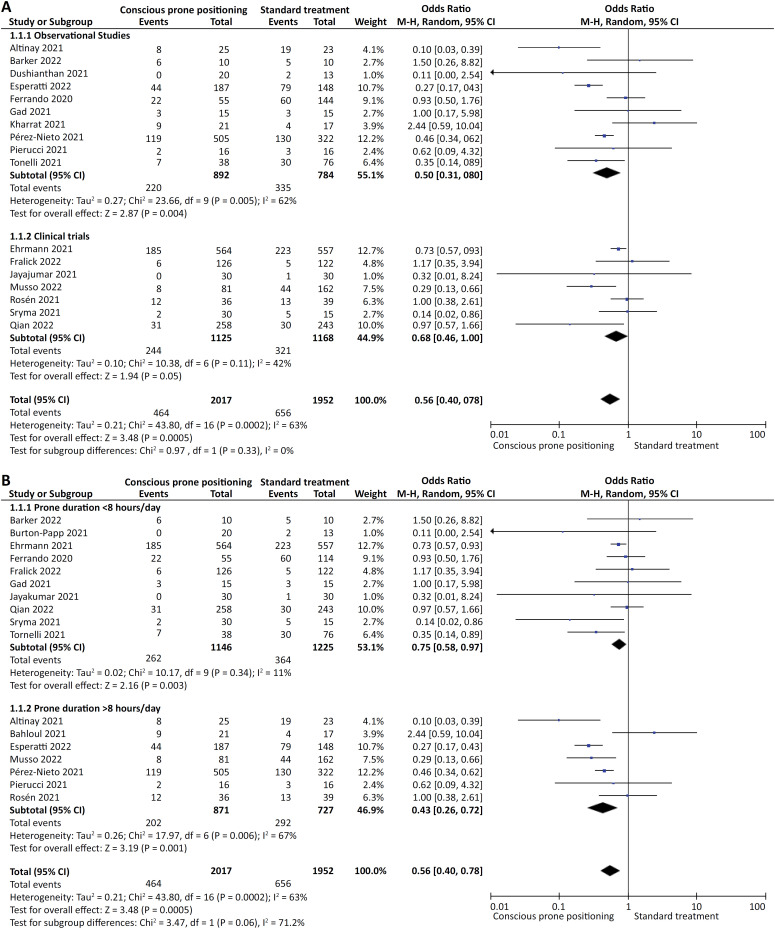

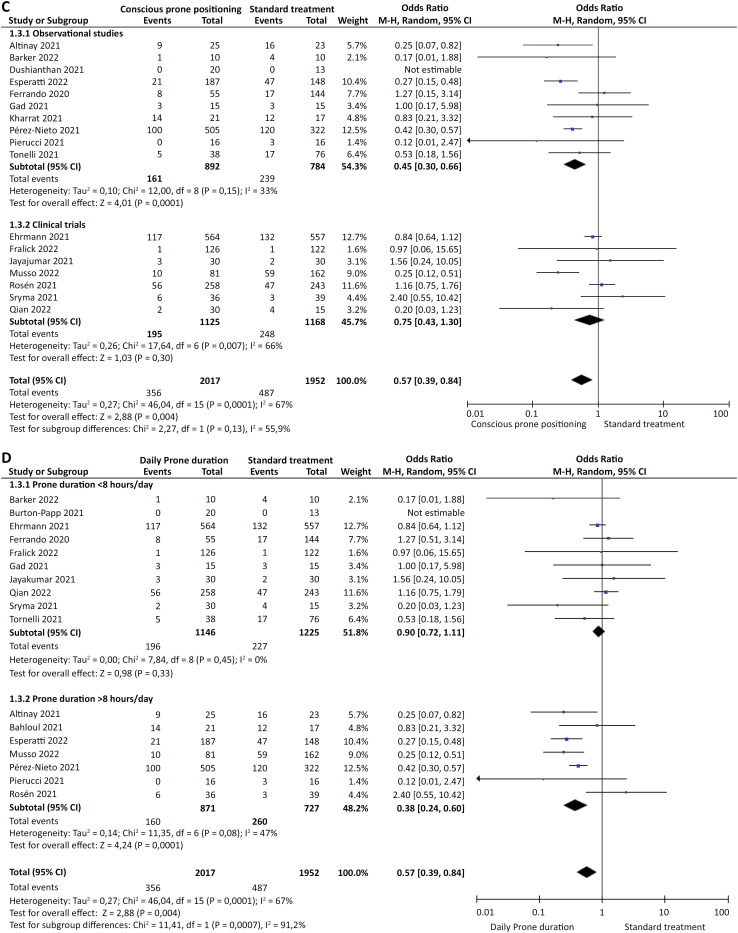

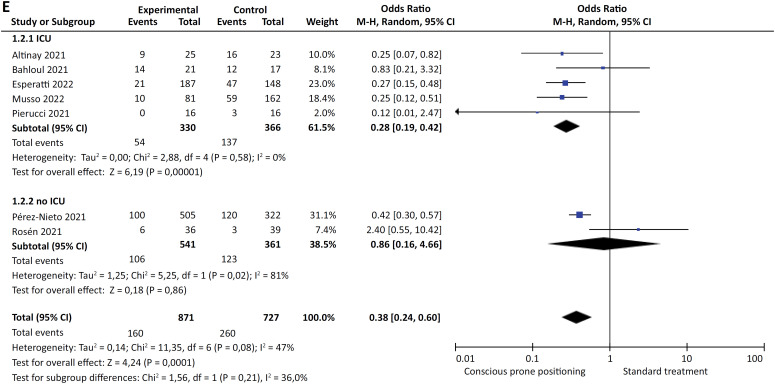
Effect of continuous prone positioning.

Nonintubated COVID-19 patients undergoing CPP had 44% lower odds of ETI (OR 0.56; 95%CI 0.40 - 0.78). In addition, heterogeneity was statistically significant (*p* = 0.0002; I² = 63%) (Figures 3A and 3B). Sensitivity analysis, excluding those studies with extreme effect sizes, showed an even better protective effect of CPP against the risk of ETI (OR 0.49; 95%CI 0.35 - 0.69).

(A) On the odds of endotracheal intubation according to the type of study (observational studies *versus* clinical trials); (B) on the odds of endotracheal intubation according to the daily prone duration; (C) on the odds of mortality according to the type of study (observational studies *versus* clinical trials); (D) on the odds of mortality according to the daily prone duration; (E) on the odds of mortality in only subgroup to daily prone duration > 8 hours and site of attendance (intensive care unit *versus* no intensive care unit).

The studies by Sryma (no data on treatment location) and Ehrmann (multitrial, only 2 randomized controlled trials were conducted in the intensive care; the unit results do not change if Ehrmann's results in France and Mexico are incorporated) were withdrawn.

95%CI - 95% confidence interval; ICU - intensive care unit.

Subgroup analysis according to the type of study (observational studies *versus* clinical trials) showed no significant differences between CPP and standard treatment on the odds of ETI (p = 0.33; I² = 0.0%) (Figure 3A).

Subgroup analysis according to the daily prone duration (DPP) showed that differences existed between the time spent in the awake prone position (p = 0.06; I² = 71.2%). Specifically, the protective effect of CPP was most pronounced among those who spent > 8 hours/day in a prone position (OR 0.43; 95%CI 0.26 - 0.72) compared to those who spent < 8 hours/day in a prone position (OR 0.75; 95%CI 0.58 - 0.97) (Figure 3B).

### Risk of mortality

Conscious prone positioning in nonintubated COVID-19 patients reduced the odds of death by 43% (OR 0.57; 95%CI 0.39 - 0.84). Furthermore, heterogeneity was statistically significant (p < 0.0001; I² = 67%) (Figures 3C and 3D).

The type of study design accounted for subgroup differences (p = 0.13; I² = 55.9%) (Figure 3C). Moreover, compared to standard care, mortality odds in the CPP subgroup were 55% lower in observational studies (OR 0.45; 95%CI 0.30 - 0.66). However, in clinical trials, there were no differences in mortality in the CPP or standard care groups (OR 0.75; 95%CI 0.43 - 1.30). Furthermore, sensitivity analysis, excluding those studies with extreme effect sizes, showed an even better protective effect of CPP against the odds of death (OR 0.42; 95%CI 0.28 - 0.63).

Subgroup analysis according to the time spent in the DPP accounted for subgroup differences (p = 0.0007; I² = 91.2%) (Figure 3D). Specifically, the protective effect of CPP on the odds of mortality reached statistical significance only in the DPP > 8 hours/day subgroup (OR 0.38; 95%CI 0.24 - 0.60) and not in the DPP < 8 hours/day subgroup (OR 0.90; 95%CI 0.72 - 1.11). Furthermore, upon analyzing the subgroup of individuals who had a DPP greater than 8 hours per day, we observed a substantial decrease in mortality rates as the level of heterogeneity decreased, particularly when stratified by the care setting (ICU *versus* non-ICU). In contrast to the non-ICU-treated group (OR 0.86; 95%CI 0.16 - 4.66, p = 0.86, I2 = 86%), wherein the effect was not statistically significant, a considerable reduction in mortality was evident within the ICU-treated subgroup (OR 0.28; 95%CI 0.19 - 0.42, p < 0.001, I2 = 0%), as shown in figure 3E.

### Certainty of evidence

We upgraded the certainty of evidence, due to the fact that all of the included studies had a low risk of bias. Indirectness (including studies comparing similar interventions, similar populations, and similar outcomes), imprecision (based on a review including 3,969 patients, 1,120 intubations, and 843 deaths), or publication bias did not affect the certainty of evidence. Conversely, we downgraded the certainty of evidence because of inconsistency (I2 > 40%) and risk of bias in non-RCT and observational studies. Overall, we assessed the certainty of evidence by using the GRADE criteria to be moderate to low.

## DISCUSSION

According to our findings, CPP reduces the odds of ETI by 44% in COVID-19 patients with ARF. Furthermore, this protective effect was most noteworthy among those who spent > 8 hours/day in the prone position compared to those who spent < 8 hours/day in the prone position. Interestingly (but not unexpectedly), this decrease in the odds of ETI is not necessarily translated into a mortality reduction, due to the fact that our meta-analysis showed that CPP reduces the chance of mortality by 43% in nonintubated COVID-19 patients. However, this protective effect of CPP on mortality was statistically significant only in the DPP > 8 hours/day subgroup and not in the DPP < 8 hours/day subset. Moreover, there was a maintenance of the decrease in the odds of mortality in the CPP and DPP > 8 hours/day subgroup and those who were also treated in the ICU, with a 72% reduction in the risk of death, thus demonstrating that the benefit of decreased mortality could be expected only in this group.

This meta-analysis demonstrated CPP benefits on robust clinical outcomes by incorporating studies such as RCTs, non-RCTs and observational studies, thereby increasing the external validity of the results collected in the RCT and their applicability and generalization in the real world, which is why we incorporated all types of designs in this meta-analysis.^(22-24)^

This meta-analysis assessed the primary outcomes, such as ETI and mortality, and not surrogate endpoints. Some meta-analyses have shown improvements in laboratory outcomes, such as oxygenation parameters and different clinical outcomes; nonetheless, their conclusions have been contradictory.^(3,18,25-27)^ Moreover, a previous meta-analysis reported a composite endpoint that combined clinical and oxygen exchange parameters, as well as adverse effects.^(27)^ Other systematic reviews concluded that CPP is safe, with a low risk of mortality and intubation. However, it is impossible to draw definitive conclusions because most of the studies that were included in these reviews did not have a comparison group.^(26,28)^ Other studies showed an improvement in oxygen saturation (SpO_2_) and the ratio of partial pressure of oxygen to the fraction of inspiratory oxygen (PaO_2_/FiO_2_) but no apparent improvement in clinical outcomes, such as intubation and mortality.^(11,15,29,30)^

Although it is true that there are meta-analyses of RCTs that reach conclusions similar to our meta-analysis regarding a reduced risk of ETI,^(16,18,19,31)^ the same cannot be said regarding mortality. For example, Weatherald et al.^(31)^ conducted a study based exclusively on RCTs, which encompassed 17 studies with a total of 2,931 patients. They observed a reduced risk of ETI (RR 0.83; 95%CI 0.73 - 0.94; I² = 0%). This trend remained consistent when analyzing subgroups based on daily prone positioning time (> 5 hours/day) (RR 0.78; 95%CI 0.66 - 0.93). However, they did not find a statistically significant reduction in the subgroup with < 5 hours/day (RR 0.92; 95%CI 0.76 - 1.12).

Interestingly, their findings diverge from our own results in regard to the place of patient care. Specifically, Weatherald et al.^(31)^ discovered that patients treated in the ICU did not exhibit a reduced risk of ETI (RR 0.86; 95%CI 0.69 - 1.08; p = 0.39; I² = 30%), as opposed to those included in studies conducted both inside and outside of the ICU (RR 0.81; 95%CI 0.69 - 0.95).

Regarding mortality, Weatherald et al.^(31)^ found no evident reduction associated with CPP (RR 0.90; 95%CI 0.76 - 1.07; I² = 0%). However, they suggest that CPP may slightly affect mortality, and its favorable impact cannot be ruled out. They interpret this phenomenon as the lower rate of ETI in patients undergoing CPP not negatively influencing mortality but potentially contributing to a positive outcome.

In contrast, Qin et al.^(16)^ incorporated 10 RCTs (2,324 patients) into their SR-Ms and systematically reviewed 4 databases to analyze whether CPP reduces the rate of ETI and mortality in patients with ARF and COVID-19. The evaluation of RCTs presents a RoB2 of some concerns or high due to performance bias (absence of blinding in patients and investigators). The meta-analysis showed a decrease in the risk of ETI in patients with CPP (OR 0.77; 95%CI 0.63 - 0.93, p < 0.001; I^2^ = 0%), thus maintaining only that measure in the subgroup of patients treated in the ICU (OR 0.74; 95%CI 0.60 - 0.91; p < 0.001; I^2^ = 0%) and in the PPD subgroup > 4 hours/day (OR 0.77, 95%CI 0.63 - 0.93; p < 0.001; I^2^ = 0%).

Cao et al.^(19)^ reported on the most current SR-Ms in this regard by incorporating 8 high-quality RCTs, although the same performance bias as was observed in the previously mentioned studies is maintained.^(16,18,31)^ They systematically reviewed 5 databases (incorporating 2,657 patients) to evaluate the efficacy and safety of CPP in patients with ARF and COVID-19. The findings showed that CPP does not reduce mortality compared to standard treatment in patients with CPP in general and in those who also used oxygen therapy with high-flow nasal cannula [HFNC] (OR 0.88; 95%CI 0.70 - 1.05; I^2^ = 0%), although they demonstrated a reduction in the ETI rate in all of the patients and in those with oxygen therapy for HFNC (OR 0.72; 95%CI 0.60 - 0.86). They performed the meta-analysis by using fixed effects, in association with the absence of heterogeneity (I^2^ = 0%), which corresponds only to the lack of statistical heterogeneity but not clinical or methodological heterogeneity, given that there is no analysis by subgroups, such as the place of care (ICU *versus* non-ICU).

Our study provides essential data in the evidence of CPP in the subgroup of patients treated in the ICU and periods of considerable prone position > 8 hours/day, wherein we showed a reduction in mortality. The reason for incorporating RCTs and observational studies is based on the fact that in the real world, we can evaluate the efficacy and effectiveness of day-to-day life activities, in addition to expanding the sample size, thus showing a robust result that improves external validity, given that the studies had a low risk of bias.^(22,23)^ One study, such as ours, evaluated the efficacy and safety of CPP and its effect on the rate of ETI and mortality, in addition to its adverse effects in patients with COVID-19 and ARF. This study meta-analyzed 22 studies (7 RCTs and 15 observational) with 5,746 patients, and it was the only study to collect the largest number of patients in an SR-Ms. Similar to our results and other publications, they found a general decrease in the ETI rate (OR 0.64; 95%CI 0.48 - 0.83; p = 0.001) that was maintained in subgroups according to design and in the subgroup with prone time > 8 hours/day (OR 0.47; 95%CI 0.25 - 0.88; p = 0.001; I^2^ = 78%). Regarding mortality, similar to our study, they found a general decrease (OR 0.61; 95%CI 0.45 - 0.81; p = 0.0003; I^2^ = 60%). When subgroups analyzed this mortality, it remained only in the subgroup according to the design (observational studies) (OR 0.44; 95%CI 0.29 - 0.66; p = 0.002; I^2^ = 61%). This group attributes this finding to the fact that existing RCTs still have consistent heterogeneity in terms of different types of treatment sites (ICU versus non-ICU), prone times, adherence, and even the type of oxygen therapy that were previously used. The subgroup of prone > 8 hours and treatment in the ICU was not reported, wherein unlike our study, we found a decrease in mortality.^(32)^

The previous authors did not explore these parameters. These studies help us in understanding that the RCT does not decrease mortality because subgroups are not assessed by place of treatment (ICU versus non-ICU), as reported by Li et al.^(18)^ When considering that ICU patients (given this environment) may have other characteristics, the explanation for our findings is thought to be: (1) in the ICU, given the monitoring of the physician/patient ratio, some complications can be detected early that warrant timely interventions and lower risks of adverse outcome; (2) greater control and adherence to the CPP process, which makes conscious patients uncomfortable; and (3) patients with greater severity who need ETI during their natural evolution and IMV wherein the prone position (as a strategy) has evidence of being an effective intervention in reducing mortality.

However, some meta-analyses have shown concordance with our results (at least in part). For example, Li et al.(18) performed a meta-analysis to synthesize the outcomes associated with CPP in subjects with COVID-19-related ARDS. They searched for observational studies (all with a control group) and clinical trials in eight databases and digital repositories. By using a random-effects model, they pooled 29 studies, ten RCTs and 19 observational studies. They reported that CPP (unlike the supine position) diminished the requirement of ETI by 16% in these patients. In addition, those patients who used advanced ventilatory assistance (i.e., HFNC or noninvasive mechanical ventilation [NIV]) at enrollment and in the ICU had a 17% lower probability of needing ETI. However, this was not the case for patients receiving standard care or in other settings different from the ICU. The researchers concluded that for COVID-19 patients with ARDS, CPP diminishes the need for ETI, especially among those requiring more sophisticated ventilatory assistance (HFNC or NIV) and those admitted to an ICU. Therefore, they recommended using CPP in COVID-19 patients with ARF requiring more advanced ventilatory aid or for those admitted to an ICU.

Fazzini et al.^(29)^ conducted a meta-analysis to evaluate the effect and timing of CPP in acute hypoxemic respiratory failure patients with ARDS or COVID-19. They systematically searched five databases and included 14 studies and 2,352 patients, 99% (n = 2,332) of whom had COVID-19. Among 1,041 (44%) patients placed in the CPP, 1,021 were SARS-CoV-2 positive. After prone positioning, they significantly improved regarding the PaO_2_/FiO_2_. In addition, those patients with COVID-19 who were placed in the prone position presented with significantly less mortality; however, the risk of ETI remained equal. In general, patients endured CPP for a median of 4 hours. The authors concluded that proning that was repeatedly applied for episodes ≥ 4 hours/day demonstrated improved oxygenation among nonintubated patients with acute hypoxemic respiratory failure. In addition, awake proning appeared to be safe; however, the effect on the ETI rate and survival remained uncertain.

Schmid et al.^(3)^ performed a meta-analysis examining the effectiveness of HFNC therapy *versus* NIV and CPP *versus* standard care in COVID-19 patients. They included five RCTs (2,182 patients) and analyzed mortality, ETI, and safety. They demonstrated uncertain results that HFNC, compared to NIV, changed mortality. Furthermore, HFNC therapy increased the rate of ETI or death, and the authors did not know if HFNC therapy diminished the risk of harm. In addition, compared to usual care, CPP decreased the risk of ETI; however, its effect on mortality appeared to be insignificant. They concluded that the certainty of the evidence was very low-moderate and that there was no robust evidence favoring HFNC or NIV; however, both strategies have a significant risk for harm. Conversely, the use of CPP likely had advantages, but the mortality risk seems to have not been affected.

Evidence suggests that CPP is feasible, practical, and safe and can be performed in different hospital settings and many parts of the world. CPP accompanied by standard treatment with high-flow oxygen therapy with various devices can achieve better results. Although research indicates that this practice is effective and reduces the ETI rate (with better results in periods greater than 8 hours/day), as well as reducing mortality in the subgroup with a time of prone > 8 hours/day and treatment in the ICU, more research is still needed to elucidate its effect on mortality.

The most important strengths of this study were the following: our search strategy was broad; we included the most recent and more significant number of studies, participants, and events than any other previous review; we included studies that specifically examined the odds of ETI and mortality and excluded studies that only evaluated intermediate outcomes; we only included studies that reported an adequate control group; and we performed subgroup analysis according to other variables, such as the clinical setting (ICU *versus* non-ICU).

Conversely, this study also had some limitations. Although we performed subgroup, sensitivity, and publication bias analyses, the source of the heterogeneity was not entirely clear. Moreover, despite including a large number of patients due to incorporating RCTs, non-RCTs, and observational studies, we must consider that there is a moderate or low level of evidence, which was mainly due to the absence of RCTs, without statistical, clinical and methodological heterogeneity. The varying definitions of the exposure, outcome, and different employed NIV strategies likely explain (in significant part) this heterogeneity. It is possible that a meta-regression analysis could further explain the origin of the heterogeneity. However, we did not perform this analysis due to the limited studies that were included.

## CONCLUSION

Our findings show that the conscious prone position decreases the odds of endotracheal intubation by 44% in COVID-19 patients with acute respiratory failure and acute respiratory distress syndrome. This protective effect is more robust in those who spent > 8 hours/day in the conscious prone position. Even more critical, the conscious prone position reduces the odds of mortality by 43% in COVID-19 patients. This impact on mortality was only statistically significant in the subgroup of patients who spent > 8 hours/day in the daily prone position and even more in the subgroup with a time of prone > 8 hours/day and treatment in the intensive care unit. These results should be cautiously interpreted because of the high risk of bias from heterogeneous randomized clinical trials, nonrandomized clinical trials and observational studies that were included.
