# Supplementary material for: Conscious prone positioning in nonintubated COVID-19 patients with acute respiratory distress syndrome: systematic review and meta-analysis
Source: Crit Care Sci. 2024 Mar 25;36:e20240176en. doi: 10.62675/2965-2774.20240176-en (PMC11098076; doi:10.62675/2965-2774.20240176-en)
Supplement: Translation

# Translation: Pronação consciente em pacientes com COVID-19 não intubados e com síndrome do desconforto respiratório agudo: revisão sistemática e metanálise

**DOI:** 10.62675/2965-2774.20240176-pt

**Authors:** Gustavo Adolfo Vásquez-Tirado, Edinson Dante Meregildo-Rodríguez, Martha Genara Asmat-Rubio, María José Salazar-Castillo, Claudia Vanessa Quispe-Castañeda, María del Carmen Cuadra-Campos

**Affiliations:** 1 Trujillo Peru Escuela de Medicina, Universidad Privada Antenor Orrego - Trujillo, Peru.; 2 Trujillo Peru Escuela de Medicina, Universidad César Vallejo - Trujillo, Peru.; 3 Trujillo Peru Escuela de Posgrado, Universidad Privada Antenor Orrego - Trujillo, Peru.

**Keywords:** COVID-19, Infecções por coronavírus, Posição prona, Posicionamento do paciente, Mortalidade hospitalar, Mortalidade

## Abstract

**Objetivo:**

Revisar sistematicamente o efeito da posição prona na intubação endotraqueal e na mortalidade em pacientes com COVID-19 não intubados com síndrome do desconforto respiratório agudo.

**Métodos:**

Registramos o protocolo (CRD42021286711) e pesquisamos quatro bancos de dados e literatura cinzenta desde o início até 31 de dezembro de 2022. Incluímos estudos observacionais e ensaios clínicos. Não houve limite de data ou idioma de publicação. Excluímos relatos de casos, séries de casos, estudos não disponíveis em texto completo e estudos que incluíram pacientes < 18 anos de idade.

**Resultados:**

Incluímos 10 estudos observacionais, 8 ensaios clínicos, 3.969 pacientes, 1.120 eventos de intubação endotraqueal e 843 mortes. Todos os estudos tinham baixo risco de viés (ferramentas *Newcastle-Ottawa Scale* e *Risk of Bias 2*). Observamos que a pronação consciente reduziu as chances de intubação endotraqueal em 44% (RC 0,56; IC95% 0,40 - 0,78) e a mortalidade em 43% (RC 0,57; IC95% 0,39 - 0,84) em pacientes com COVID-19 não intubados com síndrome do desconforto respiratório agudo. Esse efeito protetor sobre a intubação endotraqueal e a mortalidade foi mais robusto naqueles que passaram > 8 horas por dia na pronação consciente (RC 0,43; IC95% 0,26 - 0,72 e OR 0,38; IC95% 0,24 - 0,60, respectivamente). A certeza da evidência, de acordo com os critérios GRADE, foi moderada.

**Conclusão:**

A pronação consciente diminuiu as chances de intubação endotraqueal e mortalidade, especialmente quando os pacientes passaram > 8 horas por dia na pronação consciente e tratamento na unidade de terapia intensiva. Contudo, nossos resultados devem ser interpretados com cautela devido às limitações na avaliação de ensaios clínicos randomizados, ensaios clínicos não randomizados e estudos observacionais. Não obstante, apesar das revisões sistemáticas com metanálises de ensaios clínicos randomizados, devemos ter em mente que esses estudos permanecem heterogêneos do ponto de vista clínico e metodológico.

## INTRODUÇÃO

A doença do coronavírus 2019 (COVID-19) foi detectada pela primeira vez em Wuhan, China, e, depois, espalhou-se pelo mundo. De todos os pacientes infectados com o coronavírus da síndrome respiratória aguda grave 2 (SARS-CoV-2), aproximadamente 20% apresentam quadro leve que pode ou não evoluir para hipoxemia, exigindo, portanto, hospitalização.^(1,2)^ A manifestação mais grave da COVID-19 é a insuficiência respiratória aguda (IRA) devido à síndrome do desconforto respiratório agudo (SDRA).^(3)^ Aproximadamente 5% dos pacientes com COVID-19 necessitam de internação na unidade de terapia intensiva (UTI) e ventilação mecânica invasiva (VMI) devido à IRA e à SDRA.^(4-7)^

Desde 1970, a estratégia de ventilação em posição prona com VMI tem sido praticada em pacientes com atelectasia e diminuição da complacência pulmonar. Essa técnica permite o recrutamento das zonas posterobasais pulmonares, melhorando a oxigenação pulmonar^(2,8,9)^ e a relação ventilação/perfusão desses segmentos pulmonares.^(4,6,10-12)^ A posição prona é uma das poucas estratégias que demonstraram reduzir a mortalidade na SDRA. Entretanto, o tempo em posição prona e a duração do ciclo precisam ser individualizados para cada paciente, não havendo precisão em seus parâmetros específicos.

A experiência adquirida com a estratégia da posição prona em pacientes mecanicamente ventilados com SDRA sem COVID-19 foi extrapolada para pacientes acordados com COVID-19, o que demonstrou resultados promissores.^(4,13)^ Além disso, durante a pandemia de COVID-19, foi usada como estratégia ventilatória em pacientes acordados e não intubados com COVID-19. Também foi administrado em pacientes intubados com outras doenças respiratórias não relacionadas à COVID-19. De acordo com as evidências atuais, parece claro que a posição prona melhora a oxigenação gasométrica.^(14)^ No entanto, não está claro se essa estratégia diminui o risco de intubação endotraqueal (IET) ou mortalidade^(15)^ em pacientes com COVID-19 não intubados e em pronação consciente.

Até o momento, há um conjunto de evidências sobre a relação entre a pronação consciente e seu impacto no risco de IET e mortalidade, conforme explorado em ensaios clínicos randomizados (ECRs) e revisões sistemáticas com metanálises (RS-Ms).^(16-19)^ As evidências atuais sugerem que não há redução significativa na mortalidade associada à terapia com pronação consciente. É importante observar que as RS-Ms dos ECRs em questão consideram principalmente a heterogeneidade estatística, ignorando possíveis variações metodológicas e clínicas que podem contribuir para o resultado observado.

Com relação à mortalidade, a exploração de outros subgrupos, como a duração prolongada da pronação e o ambiente de assistência (por exemplo, dentro ou fora UTI), poderia desempenhar papel fundamental na elucidação dessa associação.^(18)^

## MÉTODOS

### Fontes de informação e estratégia de pesquisa

O protocolo foi previamente registrado no PROSPERO (CRD42021286711). Além disso, realizamos uma pesquisa exaustiva de artigos primários (transversais, casos e controles, coortes e ECRs) em quatro bancos de dados (PubMed®, Embase, Scopus e *Web of Science*) e na literatura cinzenta (Mednar e WorldWideScience) desde o início até 31 de dezembro de 2022. Seguimos uma estratégia PECO/PICO (população: "pacientes com COVID-19 não intubados com insuficiência respiratória aguda", intervenção: "pronação consciente", controle: "tratamento padrão", desfecho: "intubação endotraqueal" OU "mortalidade"). Combinamos termos de vocabulário livre e controlado (cabeçalhos MESH e Emtree) usando conectores booleanos (Tabela 1S - Material suplementar). Durante a realização do presente estudo, não foram feitas alterações no protocolo.

### Seleção de estudos

Coletamos documentos com texto completo e resumos. Não houve limite de data ou idioma de publicação. Excluímos relatos de casos, séries de casos, estudos não disponíveis em texto completo e aqueles que incluíam pacientes menores de 18 anos de idade. Três pesquisadores independentes e cegados avaliaram os artigos. As discrepâncias foram resolvidas por consenso ou, como último recurso, por um quarto revisor que atuou como árbitro. A figura 1 mostra o processo de seleção.

**Figura 1:** Fluxograma PRISMA 2020 do processo de seleção dos estudos primários incluídos.

ECR - estudo controlado randomizado.

### Extração de dados e avaliação do risco de viés

Analisamos os artigos segundo nossa estratégia PECO/PICO e os critérios de inclusão e exclusão. Usando uma planilha, extraímos e registramos dados referentes a autores, ano de publicação, país de publicação, tipo de estudo e número de pacientes, controles e eventos (IET e mortalidade).

### Síntese e análise de dados

Reunimos as razões de chances (RCs) usando o método de Mantel-Haenszel na metanálise. Examinamos dois desfechos primários: as chances de intubação orotraqueal e mortalidade. Além disso, devido ao fato de a heterogeneidade ser substancial (I^2^ > 40%), realizamos modelos de efeitos aleatórios e análise de subgrupo, conforme o tempo diário do paciente > 8 horas ou menos, com base no tempo total diário médio de pronação consciente na maioria dos pacientes relatados nos estudos e os locais de assistência aos pacientes (dentro ou fora da UTI).^(18)^

Avaliamos a qualidade do estudo usando a ferramenta de risco de viés da Cochrane *Risk of Bias 2* (RoB 2) para ECRs,^(20)^ o *Risk of Bias in Non-randomised Studies of Interventions* (ROBINS-I) para não ECRs e a *Newcastle-Ottawa Scale* (NOS) para estudos observacionais,^(21)^ bem como o risco de viés de publicação usando gráficos de funil.

Dois revisores independentes examinaram a certeza da evidência dos desfechos de estudo em cada desfecho de estudo com base nos critérios do *Grading of Recommendations Assessment, Development and Evaluation* (GRADE). Qualquer discrepância entre os autores da revisão foi resolvida por meio de discussão com o pesquisador principal.

## RESULTADOS

### Resultados da pesquisa e características do estudo

Identificamos 764 registros na pesquisa sistemática primária e oito registros no exame secundário. Após a eliminação de duplicatas, 572 registros permaneceram para revisão do título e do resumo. Posteriormente, excluímos 544 registros. Por fim, avaliamos 28 registros com texto completo na síntese qualitativa (Tabela 1).^(1,19-37)^ Finalmente, restaram 17 artigos para a metanálise, dos quais 10 eram estudos observacionais, 4 eram ECRs e 3 eram não ECRs (Figura 1).

**Tabela 1:** Características gerais dos estudos incluídos

Autor	País/Estudo	Participantes	Desfechos
Coppo et al.^(6)^	Itália UC, ECP	n = 46 pacientes. Ambos os sexos	IET em 18 pacientes, 5 mortes não relacionadas ao procedimento
Rosén et al.^(10)^	Suécia MC, ERC	n = 75 foram randomizados, 39 no Grupo TP e 36 no Grupo PC Ambos os sexos	13 pacientes precisaram de IET no Grupo TP *versus* 12 pacientes no Grupo PC (RR 1,01; IC95% 0,46 - 2,21)
Tonelli et al.^(13)^	Itália MC, ECR	n = 114 pacientes, 76 no Grupo TP e 38 no Grupo PC Ambos os sexos	22 pacientes morreram (17 no Grupo TP e 5 no Grupo PC); 37 pacientes precisaram de IET (7 no Grupo PC e 30 no Grupo TP)
Sryma et al.^(14)^	Índia UC, não ERC	n = 45 indivíduos (30 casos e 15 controles). Ambos os sexos	A necessidade de VMI foi maior no Grupo Controle (33,3%) do que no Grupo Pronação (6,7%)
Ferrando et al.^(33)^	Espanha e Andorra MC, ECP	n = 199 pacientes em terapia com CNAF. Ambos os sexos	82 pacientes precisaram de IET (Grupo CNAF 60, Grupo CNAF + PC 22); 17 morreram no Grupo CNAF e 8 no Grupo CNAF + PC
Dubosh et al.^(34)^	Estados Unidos UC, ECP	n = 22 pacientes. Ambos os sexos	7 pacientes precisaram de IET (5 pacientes nas primeiras 48 horas, 2 após as 48 horas seguintes), 2 pacientes morreram
Kaur et al.^(35)^	Estados Unidos UC, ERC	n = 125 pacientes. Desses, 92 receberam PC precoce e 33 receberam PC tardio. Ambos os sexos	O Grupo PC precoce teve mortalidade menor do que o Grupo PC tardio (26% *versus* 45%; p = 0,039), sem diferença na taxa de IET. No entanto, idade avançada, IET, maior tempo para iniciar a PC e uso de hidrocortisona foram associados ao aumento da mortalidade
Ehrmann et al.^(36)^	Europa, América do Norte e América do Sul MC, ERC	n = 1.126 pacientes, PC 567, TP 559. Incluídos 1.121 pacientes na análise IDT. Ambos os sexos	Falha no tratamento em 40% dos pacientes no Grupo PC e 46% dos pacientes no Grupo TP (RR 0,86; IC95% 0,75 - 0,98). RR da IET 0,75 (IC95% 0,62 - 0,91), RR da mortalidade 0,87 (IC95% 0,68 - 1,11) com PC em comparação com TP aos 28 dias de inclusão no estudo
Perez-Nieto et al.^(37)^	México e Equador MC, ECR	n = 827 pacientes não intubados nos grupos PC (n = 505) e TP (n = 322). Ambos os sexos	Menos pacientes no Grupo PC precisaram de IET (23,6% *versus* 40,4%) ou morreram (19,8% *versus* 37,3%)
Dueñas-Castell et al.^(38)^	Colômbia UC, ECR	n = 212 pacientes em TP e PC. Ambos os sexos	Mortalidade geral de 34% (73/212)
Vianello et al.^(39)^	Itália UC, ECP	n = 93 pacientes foram incluídos no estudo. Ambos os sexos	A PC foi viável e segura em 50 pacientes. Receberam IET 17 pacientes e 27 aumentaram o suporte respiratório. A taxa de mortalidade foi de 9/93. Em 41/50 dos indivíduos que passaram no estudo e foram submetidos a PC, houve benefício clínico e sobrevida sem escalonamento da terapia
Altinay et al.^(40)^	Turquia UC, ECR	n = 72. Grupo PC (n = 49), analisado (n = 25). Grupo TP (n = 23), analisado (n = 23). Ambos os sexos	Grupo PC: 9 pacientes morreram e 8 precisaram de IET (p = 0,020). Grupo TP: 16 pacientes morreram e 19 precisaram de IET (p = 0,001)
Jayakumar et al.^(41)^	Índia MC, ERC	n = 60 pacientes. PC 30, TP: 30. Ambos os sexos	Grupo PC: 3 pacientes morreram e 4 precisaram de IET. Grupo TP: 2 pacientes morreram e 4 precisaram de IET
Solverson et al.^(42)^	Canada MC, ECR	n = 17 pacientes. Ambos os sexos. UTI 12, ala hospitalar 5	7 pacientes precisaram de IET e VMI; 2 pacientes morreram na UTI após um ciclo de VMI
Gad et al.^(43)^	Egito MC, ECR	n = 30 pacientes; PC 15; VNI 15	6 pacientes precisaram de VMI (3 no Grupo PC e 3 no Grupo VNI); esses mesmos 6 pacientes morreram
Burton-Papp et al.^(44)^	Estados Unidos UC, ECR	n = 81 pacientes; 20 pacientes receberam PC associada à VNI	7 pacientes foram intubados; nenhum paciente morreu
Tatlow et al.^(45)^	Austrália UC, ECR	n = 13 pacientes. Ambos os sexos	7 pacientes morreram
Bahloul et al.^(46)^	Túnez UC, ECP	n = 21 pacientes. Ambos os sexos. Grupo PC 21, Grupo TP 17	7 pacientes morreram no Grupo PC e 5 no Grupo TP; 9 precisaram de VMI. A PC não foi associada a uma redução na mortalidade ou na taxa de VMI (p > 0,05 para ambos)
Pierucci et al.^(47)^	Itália UC, ECP	n = 32 pacientes, PC 16, VNI 16. Ambos os sexos	5 pacientes foram intubados (3 no Grupo TP e 2 no PC), e 3 no Grupo TP morreram (os mesmos precisaram de IET)
Winearls et al.^(48)^	Reino Unido UC, ECR	n = 24 pacientes com CPAP. Ambos os sexos	1 paciente recebeu VMI e 4 pacientes morreram
Musso et al.^(49)^	Itália UC, não ERC	n = 243 pacientes; Grupo PC 81, Grupo TP 162. Ambos os sexos	69 pacientes morreram, 59 no Grupo TP e 10 no Grupo PC; 52 pacientes precisaram de IET, 44 no grupo controle e 8 no Grupo PC
Aisa et al.^(50)^	Irlanda UC, ECP	n = 50 pacientes	7 pacientes foram intubados (14%). A PC foi viável em 41 (82%) pacientes e 38 (76%) pacientes relataram boa tolerância
Althunayyan et al.^(51)^	Arábia Saudita UC, ECP	n = 49 pacientes. Ambos os sexos	6 pacientes foram intubados (12,2%) e 7 pacientes faleceram (14,3%)
Qian et al.^(52)^	Estados Unidos MC, não ERC	n = 501 pacientes designados 1:1 para o Grupo PC ou Grupo TP. Ambos os sexos	Grupo PC: 31 pacientes foram intubados e 56 pacientes faleceram. Grupo TP: 30 foram intubados, 47 faleceram
Fralick et al.^(53)^	Canadá, Estados Unidos MC, ERC	n = 248 pacientes; Grupo PC 126, Grupo TP 122. Ambos os sexos	2 pacientes faleceram, 1 do Grupo PC e 1 do Grupo TP
Barker et al.^(54)^	Reino Unido UC, ECCR	n = 20 pacientes; Grupo PC 10 pacientes, Grupo TP 10 pacientes. Ambos os sexos	6 (60%) pacientes precisaram de VMI no Grupo PC e 5 (50%) no Grupo TP; 1 (10%) faleceu no Grupo PC e 4 (40%) no Grupo TP
Esperatti et al.^(55)^	Argentina MC, ECP	n = 335 pacientes; Grupo PC 187, Grupo TP 148. Ambos os sexos	44 pacientes no Grupo PC (23%) e 79 (53%) no Grupo TP foram intubados; 2 pacientes faleceram, 1 de cada grupo
Kumar et al.^(56)^	Índia UC, ECP	n = 67 pacientes	NR

UC - unicêntrico; ECP - estudo de coorte prospectivo; IET - intubação endotraqueal; MC - multicêntrico; ERC - estudo randomizado controlado; TP - tratamento padrão; PC - pronação consciente; RR - razão de risco; IC95% - intervalo de confiança de 95%; ECR - estudo de coorte retrospectivo; VMI - ventilação mecânica invasiva; CNAF - cânula nasal de alto fluxo; IDT - intenção de tratar; UTI - unidade de terapia intensiva; VNI - ventilação mecânica não invasiva; ECCr - estudo caso-controle retrospectivo; NR - não relatado.

Este estudo analisou 3.969 participantes, 1.120 eventos de IET e 843 mortes. Os estudos foram desenvolvidos em todo o mundo durante a pandemia da COVID-19 (Tabela 1) até 31 de dezembro de 2022.

### Risco de viés em estudos

Todos os estudos incluídos apresentaram baixo risco de viés (Tabela 2); por esse motivo, três estudos não randomizados foram considerados análises de ECR. O gráfico de funil não sugere um risco de viés de publicação entre os estudos incluídos (Figura 2).

**Tabela 2:** Avaliação de viés dos estudos primários incluídos

Estudo	Tipo de estudo	Local da assistência	Ferramenta	Conclusão
Rosén et al.^(10)^	ECR	UTI e enfermaria médica	RoB 2	Baixo risco
Tonelli et al.^(13)^	Estudo de coorte retrospectivo	UTI	NOS	Baixo risco
Sryma et al.^(14)^	Não ECR	Sem dados	ROBINS-I	Baixo risco
Ferrando et al.^(33)^	Estudo de coorte prospectivo	UTI	NOS	Baixo risco
Ehrmann et al.^(36)^	ECR	UTI e enfermaria médica	RoB 2	Baixo risco
Perez-Nieto et al.^(37)^	Estudo de coorte retrospectivo	UTI e enfermaria médica	NOS	Baixo risco
Altinay et al.^(40)^	Estudo de coorte retrospectivo	UTI	NOS	Baixo risco
Jayakumar et al.^(41)^	ECR	UTI	RoB 2	Baixo risco
Gad et al.^(43)^	Estudo de coorte retrospectivo	UTI	NOS	Baixo risco
Burton-Papp et al.^(44)^	Estudo de coorte retrospectivo	UTI	NOS	Baixo risco
Bahloul et al.^(46)^	Estudo de coorte prospectivo	UTI	NOS	Baixo risco
Pierucci et al.^(47)^	Estudo de coorte prospectivo	Enfermaria médica	NOS	Baixo risco
Musso et al.^(49)^	Não ECR	UTI	ROBINS-I	Baixo risco
Qian et al.^(52)^	Não ECR	UTI e enfermaria médica	ROBINS-I	Baixo risco
Fralick et al.^(53)^	ECR/EM	Enfermaria médica	RoB 2	Baixo risco
Barker et al.^(54)^	Estudo de coorte retrospectivo	UTI	NOS	Baixo risco
Esperatti et al.^(55)^	Estudo de coorte prospectivo	UTI	NOS	Baixo risco

ECR - ensaio clínico randomizado; UTI - unidade de terapia intensiva; RoB 2 - *Risk of Bias 2*; NOS - Newcastle-Ottawa Scale; ROBINS-I - *Risk of Bias in Non-randomised Studies of Interventions*; EM - enfermaria médica.

**Figura 2:** Gráfico de funil do efeito da posição prona contínua sobre as chances de mortalidade.

### Risco de intubação endotraqueal

Pacientes com COVID-19 não intubados submetidos à pronação consciente tiveram 44% menos chances de IET (RC 0,56; IC95% 0,40 - 0,78). Além disso, a heterogeneidade foi estatisticamente significativa (p = 0,0002; I² = 63%) (Figuras 3A e 3B). A análise de sensibilidade, excluindo os estudos com tamanhos de efeito extremos, mostrou efeito protetor ainda melhor da pronação consciente contra o risco de IET (RC 0,49; IC95% 0,35 - 0,69).

**Figura 3:** Efeito da posição prona contínua.

(A) Sobre as chances de intubação endotraqueal conforme o tipo de estudo (estudos observacionais *versus* ensaios clínicos); (B) sobre as chances de intubação endotraqueal conforme a duração diária da posição prona; (C) sobre as chances de mortalidade conforme o tipo de estudo (estudos observacionais *versus* ensaios clínicos); (D) sobre as chances de mortalidade conforme a duração diária da posição prona; (E) sobre as chances de mortalidade somente no subgrupo em posição prona > 8 horas por dia e local de assistência (na unidade de terapia intensiva *versus* fora da unidade de terapia intensiva).

Foram retirados os estudos de Sryma (sem dados sobre o local de tratamento) e Ehrmann (dados de vários estudos, e apenas dois estudos controlados randomizados foram realizados na unidade de terapia intensiva; os resultados da unidade de terapia intensiva não mudam se os resultados de Ehrmann na França e no México forem incorporados). IC95% - intervalo de confiança de 95%; UTI - unidade de terapia intensiva.

A análise de subgrupo segundo o tipo de estudo (estudos observacionais *versus* ensaios clínicos) não mostrou diferenças significativas entre a pronação consciente e o tratamento padrão nas chances de IET (p = 0,33; I² = 0,0%) (Figura 3A).

A análise de subgrupo conforme a duração diária da posição prona mostrou que existiam diferenças entre o tempo passado na posição prona (p = 0,06; I² = 71,2%). Especificamente, o efeito protetor da pronação consciente foi mais pronunciado naqueles que passaram > 8 horas por dia em posição prona (RC 0,43; IC95% 0,26 - 0,72) em comparação com aqueles que passaram < 8 horas por dia em posição prona (RC 0,75; IC95% 0,58 - 0,97) (Figura 3B).

### Risco de mortalidade

A pronação consciente em pacientes com COVID-19 não intubados reduziu as chances de morte em 43% (RC 0,57; IC95% 0,39 - 0,84). Além disso, a heterogeneidade foi estatisticamente significativa (p < 0,0001; I² = 67%) (Figuras 3C e 3D).

O tipo de desenho do estudo foi responsável pelas diferenças de subgrupo (p = 0,13; I² = 55,9%) (Figura 3C). Além disso, em comparação com o tratamento padrão, as chances de mortalidade no subgrupo pronação consciente foram 55% menores em estudos observacionais (RC 0,45; IC95% 0,30 - 0,66). Entretanto, em estudos clínicos, não houve diferenças na mortalidade nos grupos pronação consciente ou tratamento padrão (RC 0,75; IC95% 0,43 - 1,30). Além disso, a análise de sensibilidade, excluindo os estudos com tamanhos de efeito extremos, mostrou um efeito protetor ainda melhor da pronação consciente em comparação às chances de morte (RC 0,42; IC95% 0,28 - 0,63).

A análise de subgrupo duração diária da pronação foi responsável pelas diferenças de subgrupo (p = 0,0007; I² = 91,2%) (Figura 3D). Especificamente, o efeito protetor da pronação consciente sobre as chances de mortalidade alcançou significância estatística apenas no subgrupo duração diária da posição prona > 8 horas (RC 0,38; IC95% 0,24 - 0,60), não no subgrupo duração diária da posição prona < 8 horas (RC 0,90; IC95% 0,72 - 1,11). Além disso, ao analisar o subgrupo duração diária da posição prona > 8 horas, observamos redução substancial nas taxas de mortalidade, à medida que o nível de heterogeneidade diminuía, principalmente quando estratificado pelo ambiente de assistência (na UTI *versus* fora da UTI). Diferentemente do grupo em tratamento fora da UTI (RC 0,86; IC95% 0,16 - 4,66, p = 0,86, I^2^ = 86%), em que o efeito não foi estatisticamente significativo, observou-se redução considerável na mortalidade no subgrupo em tratamento na UTI (RC 0,28; IC95% 0,19 - 0,42, p < 0,001, I^2^ = 0%), conforme mostrado na figura 3E.

### Certeza da evidência

Melhoramos a certeza da evidência, devido ao fato de que todos os estudos incluídos tinham baixo risco de viés. A indireção (incluindo estudos que compararam intervenções semelhantes, populações semelhantes e desfechos semelhantes), a imprecisão (com base em uma revisão que incluiu 3.969 pacientes, 1.120 intubações e 843 mortes) ou o viés de publicação não afetaram a certeza da evidência. Por outro lado, rebaixamos a certeza da evidência por causa da inconsistência (I^2^ > 40%) e de risco de viés em estudos observacionais e não ECRs. Em geral, avaliamos a certeza da evidência usando os critérios GRADE como moderada a baixa.

## DISCUSSÃO

De acordo com nossos achados, a pronação consciente reduz as chances de IET em 44% em pacientes com COVID-19 e IRA. Além disso, esse efeito protetor foi mais notável naqueles que passaram > 8 horas por dia em posição prona em comparação com aqueles que passaram < 8 horas por dia em posição prona. Curiosamente (mas não de forma inesperada), essa diminuição nas chances de IET não necessariamente se traduz em uma redução de mortalidade, devido ao fato de que nossa metanálise mostrou que a pronação consciente reduz a chance de mortalidade em 43% em pacientes com COVID-19 não intubados. No entanto, esse efeito protetor da pronação consciente sobre a mortalidade foi estatisticamente significativo apenas no subgrupo duração diária da posição prona > 8 horas, não no subgrupo duração diária da posição prona < 8 horas. Além disso, houve manutenção da diminuição das chances de mortalidade no subgrupo pronação consciente e duração diária da posição prona > 8 horas e naqueles que também foram tratados na UTI, com redução de 72% no risco de morte, demonstrando que o benefício da diminuição da mortalidade poderia ser esperado somente nesse grupo.

Esta metanálise demonstrou os benefícios da pronação consciente em resultados clínicos robustos ao incorporar estudos como ECRs, não ECRs e estudos observacionais, aumentando a validade externa dos resultados coletados no ECR e sua aplicabilidade e generalização no mundo real, razão pela qual incorporamos todos os tipos de desenhos nesta metanálise.^(22-24)^

Esta metanálise avaliou os desfechos primários, como IET e mortalidade, não os desfechos substitutos. Algumas metanálises mostraram melhorias nos resultados laboratoriais, como parâmetros de oxigenação e diferentes desfechos clínicos; no entanto, suas conclusões foram contraditórias.^(3,18,25-27)^ Além disso, metanálise anterior relatou desfecho composto que combinava parâmetros clínicos e de troca de oxigênio, bem como efeitos adversos.^(27)^ Outras revisões sistemáticas concluíram que a pronação consciente é segura, com baixo risco de mortalidade e intubação. No entanto, é impossível tirar conclusões definitivas, porque a maioria dos estudos incluídos nessas revisões não tinha um grupo comparativo.^(26,28)^ Outros estudos mostraram melhora na saturação de oxigênio (SpO_2_) e na relação entre pressão parcial de oxigênio e fração inspirada de oxigênio (PaO_2_/FiO_2_), mas nenhuma melhora aparente nos desfechos clínicos, como intubação e mortalidade.^(11,15,29,30)^

Embora seja verdade que há metanálises de ECRs que chegam a conclusões semelhantes à nossa metanálise com relação a um risco reduzido de IET,^(16,18,19,31)^ o mesmo não pode ser dito com relação à mortalidade. Por exemplo, Weatherald et al.^(31)^ realizaram um estudo baseado exclusivamente em ECRs, que englobou 17 estudos com 2.931 pacientes. Eles observaram um risco reduzido de IET (RR 0,83; IC95% 0,73 - 0,94; I² = 0%). Essa tendência permaneceu consistente ao analisar subgrupos conforme a duração diária da posição prona > 5 horas (RR 0,78; IC95% 0,66 - 0,93). Entretanto, eles não encontraram redução estatisticamente significativa no subgrupo duração diária da posição prona < 5 horas (RR 0,92; IC95% 0,76 - 1,12).

Curiosamente, seus achados divergem dos nossos próprios resultados em relação ao local de assistência ao paciente. Especificamente, Weatherald et al.^(31)^ constataram que os pacientes tratados na UTI não apresentaram um risco reduzido de IET (RR 0,86; IC95% 0,69 - 1,08; p = 0,39; I² = 30%), diferentemente daqueles incluídos em estudos realizados dentro e fora da UTI (RR 0,81; IC95% 0,69 - 0,95).

Em relação à mortalidade, Weatherald et al.^(31)^ não encontraram redução evidente associada à pronação consciente (RR 0,90; IC95% 0,76 - 1,07; I² = 0%). No entanto, eles sugerem que a pronação consciente pode afetar ligeiramente a mortalidade, e seu impacto favorável não pode ser descartado. Eles interpretam esse fenômeno como a menor taxa de IET em pacientes submetidos à pronação consciente que não influencia negativamente a mortalidade, mas potencialmente contribui para um desfecho positivo.

Por outro lado, Qin et al.^(16)^ incorporaram dez ECRs (2.324 pacientes) em suas RS-Ms e revisaram sistematicamente quatro bancos de dados para analisar se a pronação consciente reduz a taxa de IET e a mortalidade em pacientes com IRA e COVID-19. A avaliação dos ECRs apresenta RoB 2 de leve a alto devido ao viés de desempenho (ausência de cegamento dos pacientes e investigadores). A metanálise mostrou redução no risco de IET em pacientes em pronação consciente (RC 0,77; IC95% 0,63 - 0,93, p < 0,001; I^2^ = 0%), mantendo essa medida apenas no subgrupo de pacientes internados na UTI (RC 0,74; IC95% 0,60 - 0,91; p < 0,001; I^2^ = 0%) e no subgrupo duração diária da posição prona > 4 horas (RC 0,77, IC95% 0,63 - 0,93; p < 0,001; I^2^ = 0%).

Cao et al.^(19)^ relataram as RS-Ms mais atuais a esse respeito, incorporando oito ECRs de alta qualidade, embora o mesmo viés de desempenho observado nos estudos mencionados tenha sido mantido.^(16,18,31)^ Eles revisaram sistematicamente cinco bancos de dados (incorporando 2.657 pacientes) para avaliar a eficácia e a segurança da pronação consciente em pacientes com IRA e COVID-19. Os achados mostraram que a pronação consciente não reduz a mortalidade em comparação com o tratamento padrão em pacientes em pronação consciente em geral e naqueles que também usaram oxigenoterapia com cânula nasal de alto fluxo (CNAF; RC 0,88; IC95% 0,70 - 1,05; I^2^ = 0%), embora tenham demonstrado redução na taxa de IET em todos os pacientes e naqueles com oxigenoterapia com CNAF (RC 0,72; IC95% 0,60 - 0,86). Eles realizaram a metanálise usando efeitos fixos, em associação com a ausência de heterogeneidade (I^2^ = 0%), que corresponde apenas à falta de heterogeneidade estatística, mas não de heterogeneidade clínica ou metodológica, uma vez que não há análise de subgrupos, como o local de assistência (dentro *versus* fora da UTI).

Nosso estudo fornece dados essenciais na evidência da pronação consciente no subgrupo de pacientes tratados na UTI e períodos prolongados de posição prona > 8 horas por dia, em que demonstramos redução na mortalidade. A razão para incorporar ECRs e estudos observacionais se baseia no fato de que, no mundo real, podemos avaliar a eficácia e a efetividade das atividades da vida cotidiana, além de ampliar o tamanho da amostra, mostrando um resultado robusto que melhora a validade externa, uma vez que os estudos apresentaram baixo RoB.^(22,23)^ Outro estudo, como o nosso, avaliou a eficácia e a segurança da pronação consciente e seu efeito na taxa de IET e mortalidade, além de seus efeitos adversos em pacientes com COVID-19 e IRA. Esse estudo realizou uma metanálise de 22 estudos (7 ECRs e 15 observacionais) com 5.746 pacientes e foi o único a incluir o maior número de pacientes em uma RS-Ms. Assim como nossos resultados e outras publicações, os autores encontraram redução geral na taxa de IET (RC 0,64; IC95% 0,48 - 0,83; p = 0,001), que foi mantida em subgrupos conforme o desenho e no subgrupo com duração diária da posição prona > 8 horas (RC 0,47; IC95% 0,25 - 0,88; p = 0,001; I^2^ = 78%). Com relação à mortalidade, semelhante ao nosso estudo, os autores encontraram redução geral na taxa de IET (RC 0,61; IC95% 0,45 - 0,81; p = 0.0003; I^2^ = 60%). Quando os subgrupos analisaram essa mortalidade, ela permaneceu apenas no subgrupo conforme o desenho (estudos observacionais) (RC 0,44; IC95% 0,29 - 0,66; p = 0,002; I^2^ = 61%). Esse grupo atribui esse achado ao fato de que os ECRs existentes ainda têm heterogeneidade consistente em termos de diferentes tipos de locais de tratamento (dentro *versus* fora da UTI), duração diária da posição prona, adesão e até mesmo o tipo de oxigenoterapia usada anteriormente. O subgrupo duração diária da posição prona > 8 horas e tratamento na UTI não foi relatado e, ao contrário do nosso estudo, encontramos uma redução na mortalidade.^(32)^

Os autores anteriores não exploraram esses parâmetros. Esses estudos nos ajudam a entender que o ECR não diminui a mortalidade porque os subgrupos não são avaliados por local de tratamento (dentro *versus* fora da UTI), conforme relatado por Li et al.^(18)^ Ao considerar que os pacientes de UTI (em função desse ambiente) podem ter outras características, acredita-se que a explicação de nossos achados seja (1) na UTI, dado o monitoramento da relação médico/paciente, algumas complicações podem ser detectadas precocemente, o que garante intervenções oportunas e menores riscos de desfechos adversos; (2) maior controle e adesão ao processo de pronação consciente, o que deixa desconfortáveis os pacientes conscientes; e (3) pacientes com maior gravidade que necessitam de IET durante sua evolução natural e VMI, em que a pronação (como estratégia) tem evidências de ser uma intervenção eficaz na redução da mortalidade.

No entanto, algumas metanálises demonstraram (pelo menos em parte) concordância com nossos resultados. Por exemplo, Li et al.^(18)^ realizaram metanálise para sintetizar os desfechos associados à pronação consciente em indivíduos com SDRA relacionada à COVID-19. Os autores procuraram estudos observacionais (todos com um grupo controle) e ensaios clínicos em oito bancos de dados e repositórios digitais. Combinaram 29 estudos, 10 ECRs e 19 estudos observacionais, aplicando um modelo de efeitos aleatórios. Eles relataram que a pronação consciente (diferentemente da posição supina) diminuiu a necessidade de IET em 16% nesses pacientes. Além disso, os pacientes que usaram assistência ventilatória avançada (ou seja, CNAF ou ventilação mecânica não invasiva [VNI]) no momento do registro e na UTI tiveram probabilidade 17% menor de precisar de IET. No entanto, esse não foi o caso dos pacientes que receberam tratamento padrão ou em outros ambientes fora da UTI. Os pesquisadores concluíram que, em pacientes com COVID-19 e SDRA, a pronação consciente diminui a necessidade de IET, especialmente entre aqueles que necessitam de assistência ventilatória mais sofisticada (CNAF ou VNI) e aqueles internados em UTI. Portanto, eles recomendaram o uso da pronação consciente em pacientes com COVID-19 e SDRA que necessitavam de assistência ventilatória mais avançada ou que estavam internados em UTI.

Fazzini et al.^(29)^ realizaram uma metanálise para avaliar o efeito e o momento da pronação consciente em pacientes com insuficiência respiratória hipoxêmica aguda e SDRA ou COVID-19. Eles fizeram uma busca sistemática em cinco bancos de dados e incluíram 14 estudos e 2.352 pacientes, 99% (n = 2.332) dos quais tinham COVID-19. Entre 1.041 (44%) pacientes colocados em pronação consciente, 1.021 eram positivos para SARS-CoV-2. Após a posição prona, eles melhoraram significativamente quanto à PaO_2_/FiO_2_. Além disso, os pacientes com COVID-19 que foram colocados em posição prona apresentaram mortalidade significativamente menor; no entanto, o risco de IET permaneceu igual. Em geral, os pacientes suportaram a pronação consciente por uma média de 4 horas. Os autores concluíram que a pronação aplicada repetidamente por períodos ≥ 4 horas por dia demonstrou melhor oxigenação entre pacientes não intubados com insuficiência respiratória hipoxêmica aguda. Além disso, a pronação consciente pareceu ser segura; no entanto, o efeito sobre a taxa de IET e a sobrevida permaneceu incerto.

Schmid et al.^(3)^ realizaram metanálise examinando a eficácia da terapia com CNAF *versus* VNI e pronação consciente *versus* tratamento padrão em pacientes com COVID-19. Eles incluíram cinco ECRs (2.182 pacientes) e analisaram a mortalidade, a IET e a segurança. Eles demonstraram resultados inconclusivos de que a CNAF, em comparação com a VNI, alterou a mortalidade. Além disso, a terapia com CNAF aumentou a taxa de IET ou morte, e os autores não sabiam se a terapia com CNAF diminuía o risco de danos. Além disso, em comparação com o tratamento padrão, a pronação consciente diminuiu o risco de IET; entretanto, seu efeito sobre a mortalidade pareceu ser insignificante. Eles concluíram que a certeza das evidências era muito baixa-moderada e que não havia evidências robustas a favor da CNAF ou da VNI; no entanto, ambas as estratégias apresentam risco significativo de danos. Por outro lado, o uso da pronação consciente provavelmente teve vantagens, mas o risco de mortalidade parece não ter sido afetado.

As evidências sugerem que a pronação consciente é viável, prática e segura e pode ser realizada em diversos ambientes hospitalares e em muitas partes do mundo. A pronação consciente acompanhada de tratamento padrão com oxigenoterapia de alto fluxo com vários dispositivos pode alcançar melhores resultados. Embora as pesquisas indiquem que essa prática é eficaz e reduz a taxa de IET (com resultados melhores em períodos diários > 8 horas), além de reduzir a mortalidade no subgrupo de pacientes em duração diária da posição prona > 8 horas e tratamento na UTI, ainda são necessárias mais pesquisas para elucidar seu efeito sobre a mortalidade.

Os pontos fortes mais importantes deste estudo foram os seguintes: nossa estratégia de busca foi ampla; incluímos o número mais recente e mais significativo de estudos, participantes e eventos do que qualquer outra revisão anterior; incluímos estudos que examinaram especificamente as probabilidades de IET e mortalidade e excluímos estudos que avaliaram apenas desfechos intermediários; incluímos apenas estudos que relataram um grupo controle adequado; e realizamos análise de subgrupo conforme outras variáveis, como o ambiente clínico (dentro *versus* fora da UTI).

Por outro lado, este estudo também teve algumas limitações. Embora tenhamos realizado análises de subgrupo, sensibilidade e viés de publicação, a fonte da heterogeneidade não ficou totalmente clara. Além disso, apesar de incluir um grande número de pacientes devido à incorporação de ECRs, não ECRs e estudos observacionais, devemos considerar que há um nível de evidência moderado ou baixo, que se deveu principalmente à ausência de ECRs, sem heterogeneidade estatística, clínica e metodológica. As definições variadas de exposição, desfecho e diferentes estratégias de VNI empregadas provavelmente explicam (em parte significativa) essa heterogeneidade. É possível que uma análise de metarregressão possa explicar melhor a origem da heterogeneidade. No entanto, não realizamos essa análise devido ao número limitado de estudos incluídos.

## CONCLUSÃO

Nossos achados mostram que a pronação consciente diminui as chances de intubação endotraqueal em 44% em pacientes com COVID-19 com insuficiência respiratória aguda e síndrome do desconforto respiratório agudo. Esse efeito protetor é mais robusto naqueles que passaram > 8 horas por dia em pronação consciente. Ainda mais importante, a pronação consciente reduz as chances de mortalidade em 43% nos pacientes com COVID-19. Esse impacto na mortalidade só foi estatisticamente significativo no subgrupo de pacientes que passaram > 8 horas por dia em posição prona e ainda mais no subgrupo com um tempo de posição prona > 8 horas por dia e tratamento na unidade de terapia intensiva. Esses resultados devem ser interpretados com cautela devido ao alto risco de viés dos ensaios clínicos randomizados heterogêneos, ensaios clínicos não randomizados e estudos observacionais incluídos.
